# Estimating and projecting HIV prevalence and AIDS deaths in Tanzania using antenatal surveillance data

**DOI:** 10.1186/1471-2458-6-120

**Published:** 2006-05-03

**Authors:** Geofrey R Somi, Mecky IN Matee, Roland O Swai, Eligius F Lyamuya, Japhet Killewo, Gideon Kwesigabo, Tuhuma Tulli, Titus K Kabalimu, Lucy Ng'ang'a, Raphael Isingo, Joel Ndayongeje

**Affiliations:** 1 National AIDS Control Programme, Dar es Salaam, Tanzania; 2 Muhimbili, University College of Health Sciences, Dar es Salaam, Tanzania; 3 Commission for Science and Technology, Dar es Salaam, Tanzania; 4 Centres for Disease Control-Tanzania AIDS, Dar es Salaam, Programme, Tanzania; 5 National Institute for Medical Research, Dar es Salaam, Tanzania

## Abstract

**Background:**

The Estimations and Projections Package (EPP 2005) for HIV/AIDS estimates and projects HIV prevalence, number of people living with HIV and new HIV infections and AIDS cases using antenatal clinic (ANC) surveillance data. The prevalence projection produced by EPP can be transferred to SPECTRUM, a demographic projectionmodel, to calculate the number of AIDS deaths. This paper presents estimates and projections of HIV prevalence, new cases of HIV infections and AIDS deaths in Tanzania between 2001 and 2010 using the EPP 2005 and SPECTRUM soft-wares on ANC data.

**Methods:**

For this study we used; the 1985 – 2004 ANC data set, the 2005 UN population estimates for urban and rural adults, which is based on the 2002 population census, and results of the 2003 Tanzania HIV Indicator Survey. The ANC surveillance sites were categorized into urban and rural areas on the basis of the standard national definitions of urban and rural areas, which led to 40 urban and 35 rural clinic sites. The rural and urban epidemics were run independently by fitting the model to all data and on level fits.

**Results:**

The national HIV prevalence increased from 0% in 1981 to a peak of 8.1% in 1995, and gradually decreased to 6.5% in 2004 which stabilized until 2010. The urban HIV epidemic increased from 0% in 1981 peaking at 12.6% in 1992 and leveled to between 10.9% and 11.8% from 2003 to 2010. The rural epidemic peaked in 1995 at 7.0% and gradually declined to 5.2% in 2004, and then stabilized at between 5.1% and 5.3% from 2005 to 2010. New infections are projected to rise steadily, resulting in 250,000 new cases in 2010. Deaths due to AIDS started in 1985 and rose steadily to reach 120,000 deaths in 2010, with more females dying than men.

**Conclusion:**

The fact that the number of new infections is projected to increase steadily to reach 250,000 per year in 2010 calls for more concerted efforts to combat the spread of HIV infection particularly in the rural areas where the infrastructure needed for prevention programmes such as counseling and testing, condom accessibility and AIDS information is less developed.

## Background

Recently, the World Health Organization (WHO), the joint united nation programme on HIV/AIDS (UNAIDS) and their partners released an updated software, the Estimations and Projections Package (EPP 2005) for HIV/AIDS [1], to estimate and project adult HIV prevalence in countries with heterosexual epidemics of HIV infection. The input to EPP is surveillance data from various sites and years showing HIV prevalence among pregnant women. The assumption is that, in these countries, HIV prevalence in pregnantwomen attending antenatal clinics (ANCs) is taken to representprevalence in all adults, male and female, aged 15–49. This key assumption is based on the comparison in a large numberof studies of HIV prevalence among pregnant women and in communitysurveys among all men and women aged 15–49 [2-9].

Usually, the dynamics of national HIV epidemics are complex and almost all HIV epidemics consist of multiple sub-epidemics. These sub-epidemicsmay affect different sub-populations, occur with different timingand severity in different geographical areas, and usually evolveat different rates. Modelling the dynamics of many complex nationalepidemics realistically requires the ability to model the individualsub-epidemics of which they are composed. For countries that have recently conducted national community based surveys, the UNAIDS Reference Group on Estimates, Modelling and Projections recommends the use of national survey derived HIV prevalence estimates to calibrate the HIV prevalence level in the EPP [1]. This should bedone separately for urban and rural areas and, if the informationis available, also by geographical regions. The prevalence projection produced by EPP can be transferred to SPECTRUM to calculate AIDS deaths [10]. We used EPP and SPECTRUM soft-wares to estimate and project HIV prevalence, number of new cases of HIV infection and AIDS deaths in Tanzania for the period ranging from 1980 to 2010 using available ANC prevalence data.

## Methods

### The EPP model

The UNAIDS/WHO Estimation and Projection Package (EPP) uses available surveillance data to estimate the time trend of adult prevalence of HIV-1 at the national level. EPP can be used for either concentrated epidemics (regarded as such if HIV infection has spread rapidly in at least one defined sub-population, but is not well-established in the general population. HIV prevalence is consistently over 5% in at least one defined subpopulation but below 1% in pregnant women in urban areas) or generalized epidemics (regarded as such if HIV infection is firmly established in the general population and HIV prevalence is consistently over 1% in pregnant women).

EPP estimates the time trend of HIV prevalence by fitting a simple epidemiological model to the surveillance data provided by HIV sentinel surveillance systems.

National HIV epidemics are usually composed of multiple epidemics in different populations and different geographic areas. To reflect this, one of the fundamental principles underlying EPP is that epidemic curves can be developed separately for different populations and then combined to produce a single epidemic curve which estimates HIV prevalence at a national level. There is no limit to the number of sub-national epidemic curves you can create as long as sufficient data exists to fit a curve for each sub-population. In generalized heterosexual epidemics the normal way to divide the populations for modelling is by geographic subdivisions such as urban and rural regions or national sub-regions such as provinces or states. Separate estimates and time trends are developed for each of these geographical units, and they are then combined within EPP to produce a national estimate for HIV prevalence and its trends over time.

In developing the approach to be used in fitting, the UN Reference group on Estimates, Modelling and Projections  has determined that a model with four parameters is well suited to fitting HIV epidemic curves. The shape of the resulting epidemic curve and the influences of these four parameters are discussed in more detail in articles available at .

Briefly the four parameters are; t0 – the start year of the HIV/AIDS epidemic, r – the force of infection. A large value of r will cause prevalence to increase rapidly while a small value will cause it to increase slowly. F0, a parameter representing the initial fraction of the adult population at risk, determines the peak level of the epidemic curve. Phi- the behaviour adjustment parameter determines how the proportion of new entrants in the adult population who are at risk changes over time. If phi is negative, people reduce their risk in response to the epidemic and the curve shows a sharper decline after peak. If phi is zero, the proportion at risk remains constant and the prevalence decline after the peak as people die. If phi positive, risk actually increases over time and prevalence falls less quickly or stabilizes at a high level.

### Spectrum

Spectrum is a modular program that is used to examine the consequences of current trends and future program interventions in reproductive health. The core of Spectrum is a demographic projectionmodel, called DemProj that projects the population by age andsex. Other modules interact with the demographic projection. They can be used as required. These modules address a varietyof issues including impacts of HIV/AIDS (AIDS Impact Model;AIM), resource allocation for HIV/AIDS programs (Goals), thecost effectiveness of programs to prevent mother to child transmissionof HIV (PMTCT), options in family planning programs (FamPlan), adolescent reproductive health (NewGen), and the consequencesof high fertility and rapid population growth (RAPID). It contains a database of population information that providesinstant access to the population estimates and projections ofthe United Nations Population Division for all countries andregions of the world. The program and manuals can bedownloaded from the Futures Group website at . In this study data from the EPP model was transferred to the spectrum for calculations of deaths arising from AIDS.

### The ANC data

**Figure 1:**
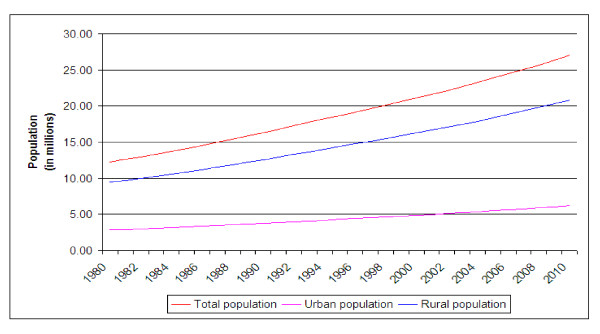
Estimates and projections of rural, urban and total adult Tanzania populations from 1980 to 2010.

The HIV prevalence data set from ANC clinics covering the period from 1985 to 2002 was agreed upon in a consensus workshop held in Tanzania in 2004 to be used for future estimations and projections. The data set was updated by incorporating the HIV prevalence data from ANC clinics for 2003/2004. A new work set for the current estimates was then created in the EPP 2005 software. In this work set a generalized epidemic state was selected and the national epidemic was defined as urban and rural sub-epidemics. The population was divided into those urban and rural areas in the proportions of 77.4% for rural and 22.6% for urban areas based on the 2002 national population census [11]. The rural, urban and total adult populations in Tanzania are shown in Figure 1.

### The ANC surveillance sites

The ANC surveillance sites were categorized into urban and rural areas on the basis of the standard national definitions of urban and rural areas. This definition led to the total number of sites being stratified into 40 urban and 35 rural clinic sites.

### Data processing

HIV Prevalence data of ANC attendees was available from 1986 to 2003 and were entered into the EPP 2005 urban and rural pages. The t0, f0, r and φ were all not fixed to allow the engine to search for the best curve fit. As recommended by the UNAIDS Reference Group on Estimates, Modelling and Projections the ANC HIV prevalence was calibrated using results of the population survey carried in 2003 that showed HIV prevalence to be 5.3% for rural and 10.9% for urban areas [12]. The ANC prevalence estimates during the same period for urban, peri-urban, rural and rural/peri-urban (a combination of rural and peri-urban) populations were 11.2%, 6.5%, .3.7% and 5.2%, respectively.

Since rural clinics include peri-urban sites, it is evident that they are not truly rural; the UNAIDS Reference Group has recommends HIV seroprevalence values from these rural sites be adjustedto 0.8 of their original value [2], to adjust for the inflating effect of peri-urban sites where HIV prevalence tends to be higher [13-19]. Deaths due to AIDS were calculated using SPECTRUM [10].

## Results

**Figure 2:**
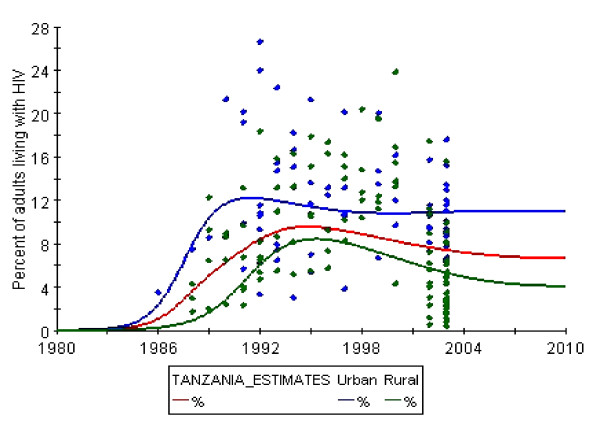
Estimates and projections of HIV infection among adult Tanzanians from 1980 to 2010.

**Figure 3:**
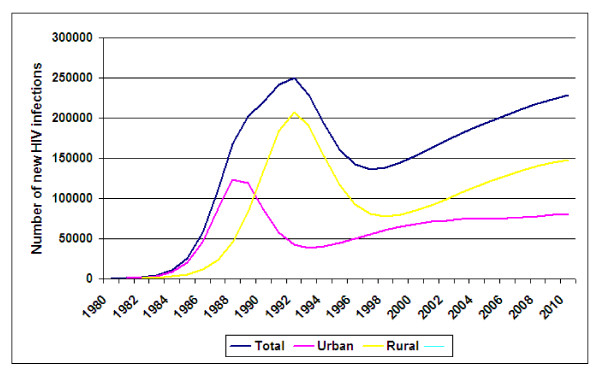
Estimates and projections of new cases of HIV infection among adult Tanzanians from 1980 to 2010.

**Figure 4:**
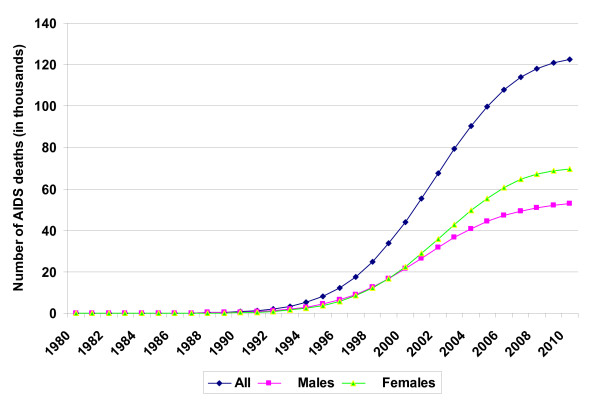
Estimates and projections of AIDS deaths among adult Tanzanians from 1980 to 2010.

Figure 2 depicts estimates and projections of HIV prevalence for the whole country as well as for urban and rural sub-populations covering the period from 1980 to 2010. The urban curve shows an increasing HIV prevalence from 0% in 1981 to a peak of 12.6% in 1992 and subsequently leveled to a plateau between 10.9% and 11.8% during the years from 2003 to 2010. The rural curve shows a steeply increasing HIV prevalence trend until 1995 when it reached its peak at 7.0% that was subsequently followed by a gradual decline to reach 5.2% in 2004, and then stabilizing at between 5.1% and 5.3% from 2005 to 2010. Fig 3 shows the estimates and projections of new HIV infections from 1980 to 2010. According to this figure, new infections rose to peak of 250,000 infections per year in 1995, before declining to 140,000 in 1997, and then steadily rising again to 225,000 new infections in 2010. Between 1983 and 1990 the number of new infections in urban areas was higher than that of rural areas. Since 1991 more new infections are encountered in rural areas, and according to estimates and projections, the absolute number of new HIV infections in rural areas will remain higher and reach twice that of urban areas in 2010 i.e 150, 000 versus 70,000. AIDS deaths started in 1985 and increased steadily to reach 120,000 in 2010, with more females dying than males (Fig 4).

## Discussion

This study presents estimates and projections of HIV infection and AIDS deaths in Tanzania, as well as for the urban and rural sub-populations for the period ranging from 1980 to 2010 (Fig 2 and 3) and AIDS deaths (Fig 4)using the EPP 2005 software [1] and the SPECTRUM [10].

There are several limitations in the EPP that need to be highlighted. One such limitation is related to the qualityand non-representative nature of data available at present. Rural data areoften not very representative of rural populations, and theEPP by itself cannot resolve this problem. We tried to minimize this bias by lowering the HIV prevalence of rural population by a factor of 20% as recommended by the UNAIDS Reference Group [1]. Furthermore, the current version of the EPP, for all its sophistication, is still only a curve fitter. It takes the data points providedand adjusts the four parameters (t_0_, r, f_0 _and φ to find single fixed valuesof those parameters that fit the observed data. This does notallow it to deal with issues such as behavioural change in responseto interventions (for example increased condom use, decreasesin STIs due to improved treatment, and changes in the size ofthe at-risk population as a result of vaccine introduction). Moreover, the choice of values of the four parameters in EPP is dependent on the user's understanding of the epidemic in the country, and may, therefore not necessarily be the best representation of the local epidemic. At present, the EPP can give no estimates of the uncertaintyassociated with model fits, nor can it estimate high and lowfuture scenarios for the HIV epidemic based on the parametersfit. Finally, as currently implemented, the EPP only allows exit from populationsthrough mortality. Thus, users must be cautionedagainst taking the model too literally.

The overall national HIV estimates, which represent an average of the rural and urban populations, depict a rise, fall and eventually stabilization of HIV prevalence, which is a natural occurrence due to mortality lagging behind initial infection by years.

When comparing the urban and rural data we found that the urban HIV prevalence peaked at 12.6% in 1992 and subsequently leveled to a plateau at around 11% while the rural HIV prevalence peaked three years later in 1995 at 7.0 and then stabilizing at around 5.2% (Fig 2). The gap between rural and urban areas does not appear to be decreasing and, according to projections, it will remain at 6%.

With regard to new HIV infections, between 1983 and 1990 the number of infections in urban areas was higher than that of rural areas. Since 1991 more new infections are encountered in rural areas, and according to estimates and projections, the absolute number of new HIV infections in rural areas will remain higher and reach twice that of urban areas in 2010 i.e 150, 000 versus 70,000. The larger absolute numbers of new infections in rural areas is due to the fact that majority (77%) of Tanzanians live in these areas. The spread of HIV infection in the rural areas needs special attention given the fact that the infrastructure needed for prevention programmes such as counselling and testing, condom accessibility and AIDS information is less developed [20]. Two types of rural areas that are particularly vulnerable to HIV are recognized as those along truck routes and those that are in areas bordering other countries, an observation which has been linked with **community factors such as the level of trade, social and economic activity **[21],**and ratio of bar workers per male population and level of community mobility **[22].

Finally, it is important to note a rise in AIDS deaths that is projected to reach 120,000 deaths per year in 2010, and that more than half of these deaths (58.3%) will occur among women.

## Conclusion

The fact that the number of new infections is projected to increase steadily to reach 250,000 per year in 2010 calls for more concerted efforts to combat the spread of HIV infection particularly in the rural areas where the infrastructure needed for prevention programmes such as counselling and testing, condom accessibility and AIDS information is less developed. Two types of rural areas that are particularly vulnerable to HIV are recognized as those along truck routes and those that are in areas bordering other countries.

## Competing interests

The author(s) declare that they have no competing interests.

## Authors' contributions

RS and GS designed the study and together with TT and NJ their supervised the field work. RI and JN performed the statistical analyses. GS, EFL and MIM prepared the draft manuscript with assistance from all authors. Finally, all authors read and approved the final manuscript.

## Pre-publication history

The pre-publication history for this paper can be accessed here:
